# The acceptability of homebased exercise snacking and Tai-chi snacking amongst high and low function UK and Taiwanese older adults

**DOI:** 10.3389/fragi.2023.1180939

**Published:** 2023-08-01

**Authors:** Ian Ju Liang, Jessica Francombe-Webb, Polly M. McGuigan, Oliver J. Perkin, Dylan Thompson, Max J. Western

**Affiliations:** Department for Health, University of Bath, Bath, England, United Kingdom

**Keywords:** exercise snacking, Tai-Chi, acceptability, physical function, older adults, cultural differences

## Abstract

**Introduction:** Exercise “snacking” and Tai-chi ‘snacking’ protocols are designed to overcome typical barriers to older adults’ participation in muscle strength and balance exercise, using short bouts of home-based exercise. This study aimed to investigate the acceptability of homebred exercise- and Tai-chi snacking in British and Taiwanese older adults of high and low physical function.

**Methods:** Thirty-three British and Thirty Taiwanese older adults took part in semi-structured interviews, after trying 1-week of exercise- and Tai-chi snacking. The interview schedule and deductive framework analysis was based on the seven components of the Theoretical Framework of Acceptability (TFA). Differences between the Taiwanese and United Kingdom participants and those considered high *versus* low physical function were also analysed.

**Results:** Both snacking regimes were found to be convenient and easy to implement. Participants reported that no activity had to be given up, and considered the programmes would be beneficial to their physical and mental health. Interestingly, more UK-based participants preferred the elegant and relaxing movements of Tai-chi snacking, yet participants with low physical function experienced difficulties when mastering Tai-chi movements. A few high physical function participants perceived exercise snacking to be tedious.

**Discussion:** Overall, the snacking exercise was found to be acceptable and useful. Personal affective attitude and different cultural backgrounds may affect exercise participation. Nevertheless, it is important to consider individuals’ physical function when designing exercise regime. The findings indicate that making Tai-chi snacking easier to master initially, building in progression and adding some upper body movements in the exercise snacking may further enhance acceptability.

## 1 Introduction

Regular strength and balance exercises can prevent a loss of physical function, maintain activities of daily living, and decrease disability or fall risk in older adults (Chou et al., 2012; de Labra et al., 2015; Bauman et al., 2016; Hamed et al., 2018). To recognize these benefits, the World Health Organization along with numerous national governing bodies recommend that older adults should engage in exercises to train strength twice per week and balance three times per week, typically advocating structured exercise sessions as the best solution (ACSM, 2018; UK Government, 2019; WHO, 2020). However, recent surveys suggest that many older adults do not meet these guidelines and research has shown that diversifying the types of structured exercise offered may benefit older adults’ physical and mental health as well as quality of life (Chen et al., 2021). For instance, there is growing evidence to support the health benefits of regular Tai-chi practice, including improved physical, cognitive, and psychological function (Chen et al., 2021). Packaging strength exercise and Tai-chi in “snacking” formats recognizes that older adults’ participation in traditional group-based exercise sessions is impacted by a variety of preventative factors that may reduce exercise and physical activity among older people. The aim of this qualitative study, therefore, is to explore older adults’ experiences of exercise snacking as a specific form of structured exercise session and to explore the acceptability of exercise and Tai-chi snacking for older adults of varying levels of physical function. Moreover, our analysis examines cross-cultural differences in terms of the experiences of older adults in the United Kingdom (United Kingdom) and Taiwan.

### Older adults’ exercise and exercise snacking

Without intervention, muscle function degenerates as people get older, increasing their risk of comorbidity, dependence and premature mortality (Pate et al., 1995; Lunney et al., 2003). Advancing age is accompanied by sarcopenia and loss of muscle size and strength, with data from longitudinal studies suggesting that muscle strength is lost at 3%–4% and 2.5%–3% per year in males and females respectively from the age of 75 onwards (Mitchell et al., 2012). Weak muscular strength and poor balance limits the ability to safely execute daily activities and can increase the likelihood of falling in older populations (Jung, 2008). Consequently, age-associated loss of physical function can place an enormous strain on health and social care systems around the world (Beaudart et al., 2014; Florence et al., 2018; Bruyère et al., 2019; Pinedo-Villanueva et al., 2019). Qualitative studies have demonstrated that older adults’ perceptions of frailty and their fear of falling can have a significant impact on wellbeing, sense of self, choice of daily activities as well as on physical health outcomes (Warmoth et al., 2016; Schoenborn et al., 2018; Van Damme et al., 2020). As the global population ages, the healthcare cost of falling is forecast to dramatically rise in the coming years (Fields et al., 2015; Government, 2020). Preserving or even improving muscle strength, balance and overall physical function is therefore an essential strategy for enhancing older adults’ quality of life and reducing the detrimental health and economic impacts of a loss of mobility.

One of the main factors associated with loss of muscle function is a reduction in physical activity, specifically exercise or movements that test one’s strength, balance, or physical function (Leveille et al., 2002; Thompson et al., 2012; Cadore et al., 2014). Focusing on older adults in the United Kingdom and Taiwan, latest data from the United Kingdom suggests that only 12% of adults aged 65, and 5% aged over 75 meet the twice-weekly muscle strengthening and balance guidelines (Department of Health, 2016). Similarly, Lin et al. (2018) found that only 22.4% of 1,068 Taiwanese older adults met both aerobic and muscle strengthening recommendations.

Exercise participation for older adults is affected by motivational, social, and environmental factors which include differences in training, motivation, exercise habits, social support, socioeconomic status, transport links and infrastructure, and neighborhood characteristics (Booth et al., 2000; Farrell et al., 2013; Jang et al., 2016). Research has found that older adults often cite lack of time, feeling lazy, apathy towards exercise, lack of self-efficacy for exercise, fear of falling, lack of access or convenient spaces for activity, insufficient support, feeling pressured in group leisure or gym settings, and economic constraints as major barriers to regular exercise participation (Lees et al., 2005; Franco et al., 2015; Burton et al., 2017a; Burton et al., 2017b; Martín-Moya et al., 2020). To overcome these barriers, supporting older adults to engage in unsupervised home-based strength and balance exercise might be a practical alternative to traditional exercise training and more acceptable to older adults with fear, discomfort, or dislike of traditional resistance exercise and gym settings (Glenn et al., 2015; Orange et al., 2019).

Several studies have found that intermittent short-bout exercises have higher adherence than long-bout exercises (Jakicic et al., 1995; King et al., 1995; Sparling et al., 2015). The term ‘exercise snacks’ has been used to describe breaking up a single session of continuous exercise into several short periods of exercise spread throughout the day (Francois et al., 2014). A daily home-based exercise snacking regime is considered to be an accessible and effective alternative to traditional resistant exercise in older adults wanting to improve their physical function (Perkin et al., 2019). However, more research is required on the subjective experiences of older adults engaged in exercise snacking to understand their experiences and ensure recommendations are meaningful and relevant to this population (Phoenix et al., 2015).

### Tai-chi as a form of exercise snacking

Tai-chi is receiving increased attention within ageing research. Originating from China in 18th century, Tai-chi is performed with slow movements and mental concentration; it is one of the traditional Chinese martial arts (Chila, 1996). Tai-chi is based on balancing ‘Yin and Yang’ energies, meaning ‘dark-bright’ or positive-negative, and is an ancient Chinese philosophy (Kuo, 2004). A number of studies have found that practicing Tai-chi positively affected balance, flexibility, lower extremity functions, and cardiovascular functions (Liu, 2008; Miller and Taylor-Piliae, 2018; Easwaran et al., 2020) and can lower the risk of falls (Li et al., 2005; Nyman, 2020; Li et al., 2021). Researchers have also found that older Tai-chi practitioners had better ankle and knee proprioception, longer single-leg stance time, stronger lower limb muscles, and better jumping ability (Mak and Ng, 2003; Gyllensten et al., 2010; Zhong et al., 2020).

As with traditional resistance training, Tai-chi is typically advocated or performed in leisure centers or settings, and in extended (i.e., 45–60 min) group-based sessions and has yet to be trialed in a ‘snacking’ format. The creation of an easy to master home-based Tai-chi snacking programme may help engage more older adults as well as improve self-efficacy and adherence. This format and structure of Tai-chi snacking marks a break in the traditional format mentioned previously that includes long, sometime expensive, group sessions (Braithwaite et al., 1998; Lo et al., 2020). Our team has studied the effectiveness of exercise snacking on leg muscle size and strength (Perkin et al., 2019), and also begun to explore the feasibility of implementing exercise and Tai-chi snacking (Liang et al., 2021). It is also imperative to explore, alongside this, the qualitative experiences of older adults themselves and whether it is an acceptable and meaningful mode of exercise within their everyday lives. It is this qualitative aspect that this paper focuses on. Before moving on to the methods and data analysis, however, we will firstly spend some time considering the unique cultural contexts of the United Kingdom and Taiwan and the theoretical framework that underpins the study.

### Exercise and ageing in the United Kingdom and Taiwan

Few studies have discussed home-based exercise participation in older populations from cross-cultural angles, as such, this paper makes a significant contribution to knowledge about cross-cultural experiences of exercise snacking acceptability that can improve older adults’ later life whilst recognizing the need for analyses that are culturally diverse and sensitive to context.

The first author grew up and has lived in Taiwan, having learnt and practiced Tai-chi for over 20 years. Upon moving to the United Kingdom, she recognized that Tai-chi was not as popular or widespread. In fact, what resonated for her were some distinct socio-cultural and spatial similarities and differences in physical activity and exercise participation in Taiwan and the United Kingdom. For example, the outdoor environment in the United Kingdom is more conducive to active commuting and the weather makes walking and being active outdoors easier and more enjoyable, whereas Taiwanese neighborhoods are busier, with more traffic congestion and the weather is a barrier to participation. Yet physical activity and exercise are engaged for similar health and body image outcomes across both countries (Giles-Corti and Donovan, 2002). The experiences of growing up, living and now researching in different countries with different values around physical activity, exercise and health have shaped the project aims as a whole. Moreover, it is these differences that also need further research as the health of ageing populations are of global concern.

### Guiding framework: the theoretical framework of acceptability

Given the social-historical differences, the distinct cultural origins of Tai-chi (i.e., East Asia) and the strength exercise snacking developed in the United Kingdom, the present research aims to explore the acceptability of strength-based exercise-snacking and Tai-chi snacking and discuss whether the acceptability of these interventions varies from British to Taiwanese populations.

The theoretical framework of acceptability presents seven components that are key to understanding acceptability of an intervention targeting health behaviors (Sekhon et al., 2017). Specifically, the framework focuses on how people feel about an intervention (affective attitude); the amount of effort the intervention would require (burden); how well the intervention relates to peoples beliefs and values (ethicality); how well the intervention is understood (coherence); whether or not other benefits need to be given up to engage with the intervention (opportunity cost); whether people believe the intervention will work for them as intended (perceived effectiveness); and an individual’s confidence in their ability perform the intervention (self-efficacy). It is through this systematic lens that the present study will explore how acceptable exercise and Tai-chi-snacking is to older adults of varying levels of physical function and whether the acceptability of these interventions differs in both Western and Eastern older adults residing in the United Kingdom and Taiwan.

## 2 Methods

### 2.1 Study design

To examine the experiences and acceptability of exercise snacking and Tai-chi snacking a qualitative study design was developed. This included a preliminary screening assessment which was used to characterize and screen participants for eligibility and demonstrate the exercise and Tai-chi snacking protocol. Participants then were asked to try out each of the exercise formats over the proceeding 7 days (i.e., 3 consecutive days of each format with a rest day in between). Subsequently, on day 8, the participants engaged in one-to-one semi-structured qualitative interviews exploring their experiences of the exercise programmes. Ethical approval for the study was provided by the University of Bath Research Ethics Approval Committee for Health (Reference: EP 18/19,107).

### 2.2 Participants

The present study recruited both male and female adults aged 65–80 years inclusive who were not regularly engaging in recreational sports or structured exercise. Participants were excluded if they: a) scored less than 4 or 0 in any sub domain of the SPPB; b) were diagnosed with and treating any chronic illness, including cardiac, pulmonary, liver, or kidney abnormalities, uncontrolled hypertension, cancer, peripheral arterial disease; c) had musculoskeletal injuries that would hamper or prevent their participation in exercise; and d) suffered from unwanted responses to exercise including chest pain, dizziness, or loss of consciousness, or have been instructed by their doctor to only do physical activity recommended by them. To enable comparisons between two distinct cultures, participants were recruited from both the United Kingdom and Taiwan. Thirty-three British and 30 Taiwanese older adults were recruited. All United Kingdom participants passed screening tests, while three of an initial thirty-three screened Taiwanese participants scored lower than 4 on the Short Physical Performance Battery (SPPB, Guralnik et al. (1994)) and were deemed ineligible for the study.

### 2.3 Taiwanese participant recruitment and protocol

The Taiwanese-based protocol was advertised by local retirement communities or carried out by word of mouth. Potential participants were provided with a participant information sheet and asked to sign an informed consent form and a screening questionnaire to ensure that participants did not exhibit any physiological condition that posed an undue personal risk.

Eligible Taiwanese participants underwent baseline eligibility screening assessments using SPPB for characterizing. Participants who scored less than 4 on SPPB or scored zero on any component of the test were excluded. Participants who scored SPPB over 8 were classified as ‘high physical function’ and who scored four to seven were in ‘low physical function group’. Participants were given an exercise demonstration on how to safely perform the exercise snacking and Tai-chi snacking activities at home. This took place in either their houses or local community centers chosen by them and were asked to record self-reported exercise logs. Participants were then asked to do 3 days of Tai-chi snacking and 3 days of exercise snacking (in a randomly assigned order) with a day off in between. Taiwanese participants underwent an in-person qualitative interview focusing on their experiences of the Tai-chi snacking and exercise snacking activities they undertook the day after finishing their trial week. 30 Taiwanese participants completed the study.

### 2.4 United Kingdom participant recruitment and protocol

To recruit United Kingdom participants the study was advertised on the University web pages and distributed to local charity organizations and social clubs with an older adult membership. Interested participants were invited to the University campus and asked to sign an informed consent form and complete a screening questionnaire. Thereafter, participants underwent the SPPB test and an exercise demonstration in the lab and, a week after trialing the exercises at home with 3 days for each exercise format and a day of rest in between, returned to the University for a semi-structured interview. A total of 34 individuals initially signed up to the study; however, only 16 participants completed the in-person assessments before United Kingdom lockdown restrictions came into effect in response to the COVID-19 pandemic in March 2020.

In response to the COVID-19 lockdown policies (i.e., staying at home, shielding for vulnerable people, social distancing), the research team received ethical approval to adapt the original study to make all procedures remote. Those 18 individuals, who did not start prior to the national lockdown were approached again and sent a revised participant information sheet to see if they would like to take part in this adapted remote study. The key adaptations that made the study COVID-safe and compliant with current regulations were.

a) all screening was conducted via online survey and an adapted video call-based assessment of physical function
b) the demonstration of exercise- and Tai-chi snacking was delivered via video calling software
c) interviews were conducted via video call.

Interested participants were sent a hyperlink directing to a web page with the participant information sheet and a consent tick box. Participants were contacted and asked their preferred time to do the physical function screening. 17 of 18 participants took up and completed the adapted remote study.

The physical function screening included the SPPB strength and balance items, using the successfully implemented protocol we tested during the initial COVID-19 lockdown (Liang et al., 2021). Safety to complete the assessment remotely was evaluated, followed by outcome assessments of strength (the number of sit-to-stands one can complete in 60 s) and balance (time one can stand on one leg, up to 60 s) only, with the requirement to score 4 on SPPB removed as participants were no longer testing gait speed as it was deemed unreliable for administration via video call. Participants were stratified as high/low physical function by 5-reps sit-to-stand (scoring low if > 13.69s and high if ≤ 13.69s) and standing on one leg (scoring low if time standing on either leg was <10s and high if ≥ 10s) in the strength and balance tests. Participants who met the inclusion criteria and scored at least 1 in both the strength and balance components of the SPPB were given a virtual exercise demonstration and underwent an observed practice by a trained instructor (first author). United Kingdom participants underwent an in-person/video calling qualitative interview focusing on their experiences of the Tai-chi snacking and exercise snacking activities they undertook the day after finishing their trial week.

### 2.5 Exercise snacking and Tai-chi snacking protocol

Both home-based exercise snacking and Tai-chi snacking programmes included five movements which focused on lower body strength and balance training (see Supplementary file A for exercise description). In the exercise demonstration session, participants were instructed how to perform exercise snacking and Tai-chi snacking movements safely at home. Particularly, researchers introduced the elements of the two different exercise protocols (such as the repetitive mode of the exercise snacking and the meditative concept of the Tai-chi) to participants and advised participants to perform Tai-chi snacking with flowing and connecting movements as well as the focus on breathing. Participants had opportunities to discuss any concerns with researchers in the demonstration session. After the exercise demonstration session, participants were asked to do 3 days of Tai-chi snacking and 3 days of exercise snacking (in a randomized order) with a day off in between. Participants were told they could complete the exercises at a convenient time of their choosing and instructed to perform each of the five movements for 1 minute with 1-min rest in between. In the exercise snacking programme, participants were advised that the aim was to complete as many repetitions as possible in the minute. In Tai-chi snacking programme, participants were advised to perform the movements as accurately and gently as possible. Exercise written instructions (Supplementary file A) and video instructions on YouTube were provided.

### 2.6 Interview

The semi-structured interview topic guide was created based on the seven dimensions of the theoretical framework of acceptability (TFA; Sekhon et al. (2017)). Specifically, questions explored affective attitude, e.g., ‘How did you feel about the exercise snacking/Tai-chi before/during doing it?‘; burden, e.g., ‘How did you find doing the exercises every day?‘; opportunity costs, e.g., ‘Do you have to sacrifice or give up doing something for the exercises?‘; perceived effectiveness, e.g., ‘What improvements did you expect to see from these exercises?’ and ‘Do you feel that the exercises themselves had or could have any effect on you?‘; intervention coherence, e.g., ‘Was there any part of the exercises that you found particularly difficult to understand or perform?‘; ethicality, e.g., ‘Do you think that exercise snacking and/or Tai-chi snacking are appropriate and suitable for you and people in your age group?‘; self-efficacy, e.g., ‘Do you think you would carry on doing the exercise snacking and/or Tai-chi snacking on your own in the future?‘. The interviews of Taiwanese participants were conducted in Mandarin using the same topic guide, which was translated and verified by a Mandarin speaking academic.

The semi-structured interview guide allowed participants to speak freely and they were encouraged to answer the questions in as much detail as possible. To comprehensively understand participants’ experiences, additional probing questions (e.g., ‘could you elaborate?’) were added when necessary. All interviews were conducted by the first researcher and audio recorded with participant’s consent.

### 2.7 Data analysis

Digital recordings of interviews were transcribed verbatim in Microsoft Word in an anonymized format and then uploaded to QSR NVivo12 for coding and data organization. The Taiwan-based interviews were transcribed and coded in Mandarin, and then translated into English for illustration of quotes. Data were analyzed using a deductive framework analysis (Ritchie et al., 1994) aligned to aspects of the Theoretical Framework of Acceptability (Sekhon et al., 2017) and an inductive thematic analysis (Clarke and Braun, 2014) to gather any other relevant information regarding barriers and facilitators to participation, future motivation, and any opportunities to refine or develop the exercise and Tai-chi snacking protocols. This data analysis was primarily conducted by the first author (IJL); however, two further authors with qualitative research experience (JFW and MJW) checked the themes for trustworthiness and accuracy of meaning. Differences between the Taiwanese and United Kingdom participants and those considered high *versus* low physical function were also explored and highlighted where relevant.

Within the remainder of this paper, we critically examine the qualitative data from the older adult participants and their experiences of exercise and Tai-chi snacking. The discussion will be structured thematically in line with the 7 domains of the theoretical framework of acceptability (Sekhon et al., 2017). Guided by the thematic analysis (Clarke and Braun, 2014), emerging sub-themes related to cultural context and older adults’ functional movement will also be explored.

## 3 Results and discussion

**FIGURE 1:**
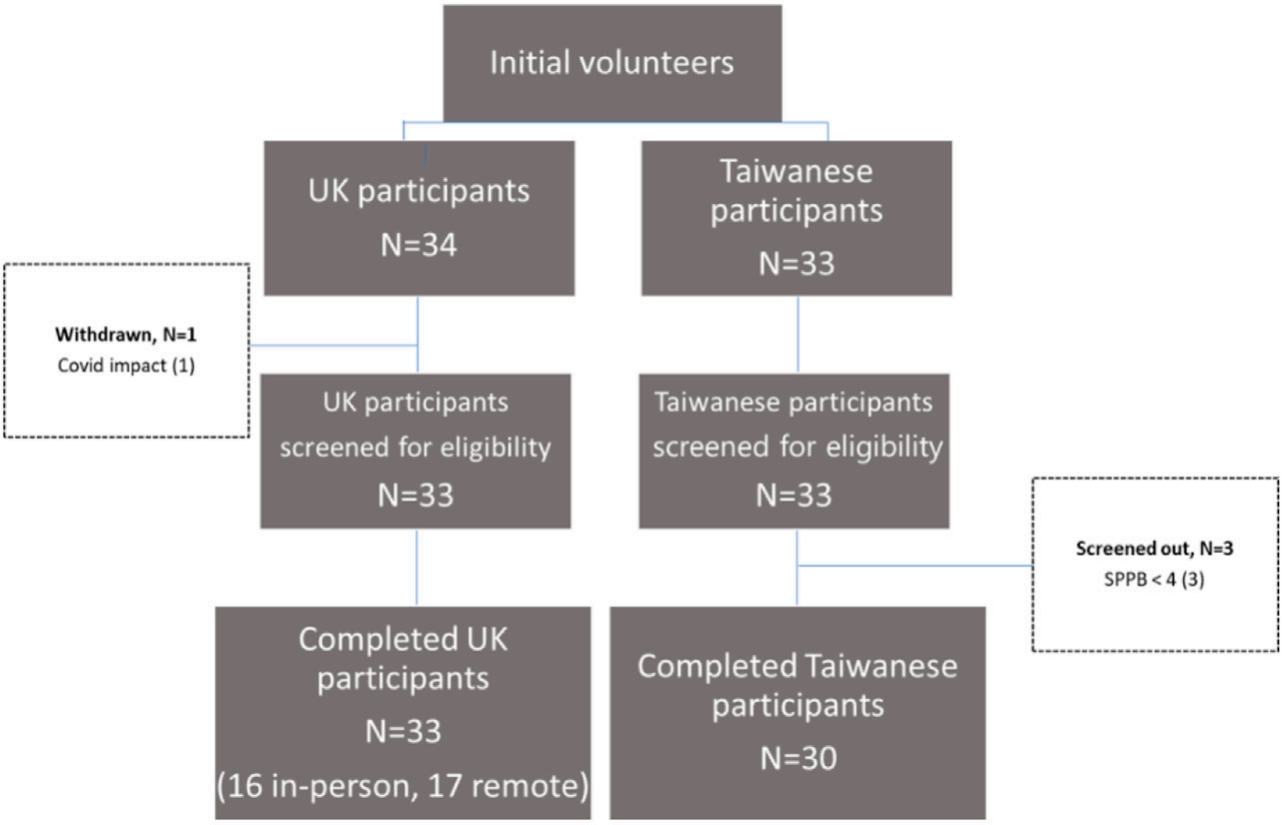
Flow diagram of participation throughout the study.

Figure 1 indicates the flow of participants through the study. There were 34 initial United Kingdom volunteers and 33 Taiwanese volunteers. One United Kingdom volunteer withdrew due to COVID-19 symptoms and 33 underwent and passed the eligibility screening tests. Of the 33 Taiwanese participants who underwent the eligibility screening tests, 3 of them were excluded due to low fitness function. A total of 63 participants completed the study. Characteristics of included participants are shown in Table 1. Compared to United Kingdom participants, the Taiwanese sample had a higher proportion of female and employed participants, were slightly younger, had fewer living alone, and had lower educational attainment. Of the United Kingdom participants, 19 were considered to have high physical function and 14 low physical function, whereas the Taiwanese sample comprised 22 participants with high physical function and 8 with low physical function.

**TABLE 1:** Demographic characteristics for United Kingdom and Taiwanese participants.

	Total	United Kingdom	TW	*p*-Value
	N = 63	N = 33	N = 30	
Female, n (%)	43 (68)	19 (58)	24 (80)	.056^a^
Age, mean ± SD	72.7 ± 4.8	73.7 ± 3.9	71.6 ± 5.5	.002^b^
Living alone, n (%)	14 (22)	12 (36)	2 7)	.005^a^
Marital status, n (%)				.279^a^
Married/civil part	45 (71)	21 (64)	24 (80)	
Divorced/Separated	3 5)	3 9)	0 0)	
Widowed	9 (14)	5 (15)	4 (13)	
Single	6 (10)	4 (12)	2 7)	
**Employment, n (%)**				.047^a^
Retired	52 (83)	30 (91)	22 (73)	
Doing unpaid work	6 9)	3 9)	3 (10)	
Still working	5 8)	0 0)	5 (17)	
**Educational status, n (%)**				<.001^a^
Secondary Education	22 (35)	2 6)	20 (67)	
Post-Secondary	8 (13)	4 (12)	4 (13)	
Vocational Qualification	5 8)	3 (10)	2 7)	
Undergraduate Degree	17 (27)	14 (42)	3 (10)	
Post-graduate Degree	11 (17)	10 (30)	1 3)	
**Physical function, n (%)**				.108^a^
High^c^	42 (67)	19 (58)	23 (77)	
Low^d^	21 (33)	14 (42)	7(23)	

^a^ Differences between groups were analysed using Chi-square tests.

^b^ Analysed using independent-samples *t*-test.

^c^ High physical function: Participants scored over 8 on SPPB, or for those who undertook the tests remotely, doing 5-reps sit-to-stand ≤13.69s and standing on one leg ≥10s.

^d^ Low physical function: Participants scored four to seven on SPPB, or for those who undertook the tests remotely, doing 5-reps sit-to-stand >13.69s and standing on one leg <10s.

The results are based on the analysis of 63 interviews of the United Kingdom (United Kingdom) and Taiwanese (TW) participants. Participant quotes are presented along with individual characteristics [Country + study ID; age; physical function category; sex].

### 3.1 Affective attitude: participants’ feelings and attitude towards the intervention

The participants’ affective attitudes highlighted how they felt about taking part in the exercise and Tai-chi snacking exercises. The United Kingdom participants expressed an interest and willingness to explore alternative training methods that differed from their previous experiences. Tai-chi was of particular interest to the participants for whom this was a completely new form of physical activity. A female participant from the United Kingdom, who engaged in other forms of exercise, remarked on this:

Well, I was interested. I thought it was a programme of exercise which I did not have. I’ve always done some exercise, but mainly flexibility exercise. And trying to tune up muscles as it were but never anything in an organized way… So that’s my basic reaction to my opinion before I tried. so I was pleased to have an opportunity to actually become more organized [UK028, 69 years, low function, female]

For others it was the combination of the exercise snacking format and Tai-chi as a form of body movement that was appealing, one United Kingdom male commented that he was ‘intrigued’ to find out what exercise snacking meant and what benefits could it bring:

I was intrigued to find out what benefit I would get from it. Um, something that I was not familiar with. I realised what was involved with the exercise snacking and I can understand that the Tai-chi was something I’d never known before. And I’d only seen it on the television, somebody waving their arms slowly, and I thought, well, it is got to be good. So, give it a try [UK006, 73 years, high function, male]

Interestingly, following engagement with both the exercise and Tai-chi snacking protocols, a number of UK-based participants expressed that they preferred Tai-chi snacking. The reasons for this varied, from enjoyment in new ways of moving the body that were felt as graceful, expressive, and relaxing to enjoying a new form of training and using muscles they rarely used. Research suggests that pleasure and enjoyment are often under-researched and under-theorized components within health studies and older adults (Phoenix and Orr, 2014). It is therefore important to identify these moments of pleasure and the multiple forms of enjoyment the participants experienced when they engaged in the snacking activities (Kirkland et al., 2011) because ‘enjoyment and pleasure are a central argument for maintaining people’s habit of health behaviors (Phoenix and Orr, 2014, p. 94) (Crossley, 2006).

Some of the participants derived enjoyment and simply from being active, feeling the sensations of their moving bodies. Others enjoyed the sense of skill mastery and/or acquisition that learning new activities offered. For participants with low physical function, Tai-chi snacking was experienced as relaxing and had the benefit of not requiring strenuous exertion:

I’m quite keen to master the Tai-chi bits… I felt very relaxed and I really enjoyed it. I felt like my whole body was moving, but no sort of muscle strain. They’re not strenuous exercises… I think that’s better really because I do not suffer from muscle fatigue the next day [UK022, 76 years, low function, male]

Alternatively, a high functioning female participant enjoyed the way her muscles were activated in ways that felt novel and were ‘quite fun’:

I thought a couple of them (Tai-chi snacking) were quite fun. It worked muscles that I do not normally use, particularly I thought the snake creep through the grass one because that was like squatting, and it worked the muscles of the front to my thighs, which do not get much use and they’re not my best muscles [UK033, 67 years, high function, female]

Nevertheless, studies have demonstrated that people tend to become bored with repetitive activities after a time, the simplicity and lack of variation are common barriers to adherence (Resnick and Spellbring, 2000; Hinman, 2002; Chen et al., 2017). These are consistence with the findings in the current study that some participants with high physical function expressed. For these participants the exercise snacking programme was too short and easy which they would not carry on doing it because of being bored and lacking interest:

I do not think I’ll keep doing it (exercise snacking) after your study. It is a bit easy and I think doing each movement for 1 minute is not enough for me [TW019, 79 years, high function, female]

…I think I might get bored (doing exercise snacking). The exercise stacking is not strenuous enough [UK013, 79 years, high function, male]

Overall, being interested in learning new form of exercise was indicated by most participants before they tried the snacking protocols, feeling enjoyment, being energized, and well-satisfied were experienced after they did the protocols. Interestingly, there were differences between participants’ preferred snacking exercises, depending on different physical functions and nationalities. More UK-based participants preferred Tai-chi snacking, citing its’ elegant and relaxing movements as a core reason. For participants with low physical function, Tai-chi snacking was gentler and less physically effortful. Few participants expressed that they might get bored of the repetitive exercise snacking movements if they were to do the programme for longer term especially for participants with high physical function. Therefore, targeting those who would benefit most with the snacking exercises is important, as well as tailoring the exercise intensity and allowing for progression or adaptations to make it optimally challenging for those who feel more capable (Teixeira et al., 2012).

### 3.2 Burden: the perceived amount of effort that required to take part in the intervention

The differing nature of the two snacking protocols were not only highlighted by the participants but they remarked on how demanding they were, both physically and cognitively. Both exercise and Tai-chi snacking were reported as being physically demanding. For these two low functioning participants, one in Taiwan and the other in the United Kingdom, the exercise snacking especially left them breathless and tired but feeling stronger:

Exercise snacking was good and some of it were strenuous. I was a little bit breathless possibly after doing the exercise snacking [TW022, 79years, low function, male]

The exercise snacking ones, I really did feel that my leg was 100% strengthening, it was not hurting. The Tai-chi ones where I was lifting my leg and arms like that. (stand on one leg and front heel kick), I did find it very tiring… [UK026, 77years, low function, female]

The female participant from the United Kingdom here identified an increase in strength as a perceived outcome from their participation. This perceived physiological impact can also have a positive impact on older adults’ wellbeing as they feel confident that they had the capability to complete everyday tasks. Likewise, studies have shown that the feeling of confidence and sense of accomplishment are important when engaging in physical activities and would lead to beneficial health outcomes (Codina et al., 2020; Molina and Myrick, 2021).

Most participants found that Tai-chi snacking needs more cognitive effort than the repetitive exercise snacking and required multitasking as participants had to memorize the movements, concentrate on breaths, and maintain balance at the same time. A few participants described Tai-chi snacking as a coordinated training because of the combination of upper and lower body movements:

I think your brain when you’re learning it needs to be engaged a lot more with Tai-chi than it does with the exercise snacking. It took a bit more concentration for me to do [UK034, 72 years, low function, female]

Specifically, participants with low physical function reported that following the programmes takes more physical effort and some struggled with continually doing the movements for 1 minute:

I found it quite tiring because I’m not used to it. I’ve had to get support by touching a table or a chair. And I found it quite difficult to balance… I did it when I felt like it because I struggled to continue to do it for 1 minute [UK011, 75 years, low function, male]

The Tai-chi ones where I was lifting my leg and arms like that. (stand on one leg and front heel kick), I did find it very tiring… [UK026, 77 years, low function, female]

For some participants time was a factor, they remarked that learning Tai-chi was time consuming but demonstrated that although time intensive, they believed that this would not persist beyond the initial phase of ‘trying to learn what I’m supposed to do’:

The exercises themselves did not take lots of time, but reminding myself about the Tai-chi, because it was only 3 days. In the long term that would not be an issue [UK033, 67 years, high function, female]

It took too long to do the Tai-chi, because I was spending hours looking at video instructions… But that’s only because I’m trying to learn what I’m supposed to do [TW021, 66 years, low function, female]

When it comes to cross-cultural comparison in the burden dimension, no differences were found between the United Kingdom and Taiwanese participants. Although Tai-chi is a common exercise form and has been part of recreations in Taiwan, the perceived effort in Taiwanese participants did not differ from the United Kingdom participants.

Taken literally, both exercise and Tai-chi snacking required physical effort, yet both of the protocols did not seem overly burdensome:

I find it really straightforward and not strenuous. You do have a break, you know, which is good [UK003, 78 years, high function, female]

It is not tiring. And you do not have to spend too much time on doing it. I mean, it only takes 10 min [TW030, 68 years, high function, female]

However, some participants with lower physical function indicated that these snacking exercises were a bit overly physically demanding, suggesting gentle progression to get up to the 1-min protocol may be needed. Similarly, Tai-chi snacking was reported to require more cognitive effort and was time consuming for some beginners. Supportive guidance to help them mastering the movements would seem necessary to reduce this burden.

### 3.3 Opportunity costs: the extent to which benefits, profits or values need to be given up engaging with the intervention

The qualitative interviews focused the lived experiences of older adults engaging in exercise and Tai-chi snacking. The notion of sacrifice came to the fore, and whether participants had to sacrifice doing their other activities for the exercise and Tai-chi snacking programmes. Most participants did not have to give up or sacrifice their time to incorporate the snacking exercises into their daily lives. This is probably a strength of the simple mode of snacking exercises that do not require lots of time and does not require people to go to a gym. The findings indicate that snacking exercises are acceptable and achievable exercise routines:

Certainly 10 min per day… I do not have to give up doing anything. Also, I have plenty of time. I can’t make any excuses about not having time… You can do them anytime and I usually did them while watching TV [TW016, 66years, high function, female]

You can fit it in with other tasks you have to do at home, like clean the room and other things outside of the door… [UK011, 75 years, low function, male]

I did not have to stop doing anything else to do it because I have the time. I mean, 10 min is not much to the day and I can fit 10 min in every day. and you do it at home, you do not need any special equipment [TW020, 77 years, low function, female]

It is easy to do, you do not have to get dressed. You do not have to go to the gym. You do not have to go through a real procedure… You can always do bits of it sitting in the chair and things in anytime. You can do these exercises in the privacy of your own home. You can do it in 5, 10 min here or there [UK002, 78 years, low function, male]

A number of participants proposed that building new habits, fitting the exercises into their routine, having self-discipline, being prompted to exercise and for some, having rewards or motivations were necessary and would be supportive for them to carry on doing the programme. According to previous studies, individuals with higher conscientiousness and self-discipline tend to engage in healthy habits (Bogg and Roberts, 2004; Kern and Friedman, 2008). The notion of habit emerged from this high functioning United Kingdom participant:

I think you just go and get it into the habit. It is remembering and having a time; it is trying to organize a time when you do it. You have to sort of discipline yourself to do it on a regular basis [UK003, 78 years, high function, female]

Homebred snacking exercise is designed to capture those individuals who do not engage in those social or group-based activities. A social setting can become a barrier to participation for individuals who are pre-frail, started to become frailer, and/or have lost confidence to go out and engage with leisure settings (Yardley et al., 2006; Biedenweg et al., 2014). Yet the fact that the exercise snacking protocol took place in individual homes had an impact on this participant’s engagement, they felt that exercise and a sense of social cohesion and interaction was an important motivator according to their previous experiences. Without ‘friends’ to ‘socialize a bit’ with, they commented that their motivation for the exercise snacking was reduced:

I believe that these exercises are good for my physical health, but I’d prefer joining group classes. You know, you can make friends and socialise a bit. Doing exercises on my own at home. sometimes I just felt lazy and did not want to do them… [TW004, 67 years, high function, female]

Research has found that when it comes to the preferred mode of exercise programmes, it is personal and varies between individuals (Franco et al., 2015; Sandlund et al., 2018). Therefore, it seems prudent to understand the preferences for social exercise, and ensure that home-based strength and balance interventions are not implied to displace other forms of group-based exercise.

Generally, participants acknowledged the snacking exercise only takes 10 min per day and does not need any equipment which is easy and convenient to do at home. The participants reported that no activity had to be given up engaging in our home-based snacking exercise programmes. We suppose that the convenience of our home-based snacking exercise is applicable and suitable for older people and this does not vary from British to Taiwanese populations.

### 3.4 Perceived effectiveness: the effectiveness participants expect to receive from the intervention and whether they consider it to be good for them

Perceived effectiveness was explored in relation to participants’ views or beliefs about the physical or mental health benefits of exercise and Tai-chi snacking. The expected and the experienced effectiveness were positive overall. In particular, participants perceived that the snacking exercises could improve their muscle strength and balance. These two participants who lived in the United Kingdom and have low functionality, articulated explicitly these perceived improvements in their balance, strength and mobility:

I think it [Tai-chi snacking] is good for me. I think probably it is good for balance. I felt my balance was getting better through it. And the snacking one [exercise snacking] I felt it is good for me, for my bottom part, lower body part. I think it probably does improve strength [UK019, 72 years, low function, female]

Well, the surprise is that I can lift my legs up higher than I did before I started. That’s an improvement. The main improvement is that I’m able to jump up, go out and start walking down the street with no aches or walking up and down stairs [UK011, 75 years, low function, male]

Studies have shown that motivators to exercise participation in older adults included preventing physical function degeneration, reducing risk of falls, improving muscle conditions, living independently, and having better wellbeing (Franco et al., 2015; Burton et al., 2017a). These are findings that cohere with the experiences of the participants in this study. When interviewed, the participants were referring to keeping up the snacking protocol going beyond the study as they considered being physically active and maintaining their mental health to be important. For one female participant the COVID-19 global pandemic and associated lockdowns have brought these concerns to the forefront, meaning she is ‘very keen’ to continue undertaking the snacking protocol:

The thing is that we’ve been isolating since March, we’re not going out. And I’m quite worried about our physical fitness and mental fitness. I think because exercise helps to stop getting Alzheimer’s. And if you’ve got memory loss, it improves it because of the blood supply so I’m very keen [UK026, 77 years, low function, female]

Similarly, other participants anticipated that continued exercise and Tai-chi snacking could improve their, already high, physical function and prevent physical and cognitive functions degeneration:

I’m aware that I’m getting older and less fit, and I needed some extra help, perhaps [TW009, 67 years, high function, male]

This may allay fear… like fear of falling, fear of getting dementia, you know, all this kind of getting-old fears that you’re doing something to help yourself [UK033, 67 years, high function, female]

In addition to these perceived effects, other participants also expressed that their mental wellbeing, cognitive condition, memory, and even sleeping quality was enhanced, even after just a few days of exercise:

I feel more flexible than before. I think these exercises ease my stiffness on my joint. My sleeping quality has improved. And because I enjoyed the exercises, I think it is good for mental health [TW007, 69 years, high function, female]

Some participants described that feeling their muscles working motivated them. Specifically, some participants revealed that Tai-chi snacking was felt to be beneficial for their balance and overall physical functions as well as their mental health. Some reflected that their flexibility and posture were better after practicing Tai-chi snacking. Participants also expected that the snacking exercises, especially Tai-chi snacking, would be beneficial for their coordination. These effects are similar to the findings from existing literature which demonstrated that practising Tai-chi can improve muscle strength, balance, mobility, and flexibility (Son et al., 2016; Chen, 2019), and is good for brain function and mental health (Chen et al., 2002; Wayne et al., 2014).

These exercises will increase my stamina… there would be flexibility and strength as well [UK023, 73 years, high function, female]

I think Tai-chi is good for coordination because you need to wave your hands and move your feet at the same time [TW010, 75 years, high function, male]

I think practicing Tai-chi is good for my balance and can strengthen my thigh muscles… I felt brighter after doing Tai-chi snacking and my sleeping quality had gotten better [TW017, 66 years, high function, female]

Like the effects of Tai-chi snacking mentioned above, Gryffin et al. (2015) reported that psychological benefits and improvement of balance are identified as facilitators to Tai-chi. However, some considered lack of awareness of benefits to be a barrier (Lo et al., 2020). To some people, it is the unknown and unfamiliar that impacted their experiences of Tai-chi. This lack of familiarity may have shaped their interpretation of the activities and the practitioners too (Gryffin et al., 2015). Since Tai-chi is relatively new in countries such as the United Kingdom, and given there is insufficient knowledge of it, UK-based participants specifically thought that introducing the principles and the background of Tai-chi was needed:

I think Tai-chi is probably very beneficial based on my knowledge of what happens in Asian side. But I think we need a clearer and longer introduction to it to make it possible to do it in any effective way [UK010, 77 years, low function, male]

The Tai-chi is lovely and if I knew more about it and could do it better, I think it would be fine… I think I prefer the Tai-chi, because it is more peaceful and more restful. probably very good for your mental health [UK024, 72 years, low function, female]

Nevertheless, most participants indicated that the 1-week trial was too short to see any significant improvement or changes and believed that the positive effects would be more long-term. Altogether, participants considered that the exercise and Tai-chi snacking would be of physical, mental, and cognitive health benefit to them. Indeed, the participants especially those UK-based were keen to learn more about the origins and values of Tai-chi and thought that this introductory information would enhance their experiences of Tai-chi snacking.

### 3.5 Intervention coherence: the extent to which the understanding of the intervention and the capability of executing it

Here we focused on interpreting how confident participants were that they understood the exercise and Tai-chi snacking programmes and how the snacking exercises worked. In general, exercise snacking movements were more coherent and easier to perform than Tai-chi snacking movements. Participants found exercise snacking to be straightforward to learn, when compared to Tai-chi on the basis that it was less complicated and time-consuming as movements did not need to be learnt and/or memorized. On the other hand, some found Tai-chi snacking more peaceful and had less pressure to perform. Nevertheless, there were not many differences between the United Kingdom and Taiwanese participants.

The exercise snacking, I found um. I mean I had no problems doing it. They are relatively straightforward. And I found the Tai-chi much more difficult [UK002, 78 years, low function, male]

The snacking bit was quite easy, but with the Tai-chi… I found it a bit hard to learn and some movements were complex. Actually, I spent lots of time watching your video and tried to get to do it (Tai-chi) on the first 2 days [TW001, 70 years, high function, female]

Personally, I enjoyed the Tai-chi. I felt more relaxed. The exercise snacking is a bit of challenge. I think because my joints are stiff. But Tai-chi was quite relaxing [TW019, 79 years, high function, female]

I would really like to say I prefer doing Tai-chi. I think it is less pressure on your knees. And the other one (exercise snacking). It puts pressure on the knees [UK014, 71 yeaear, high function, female]

For several participants, difficulty executing certain exercises arose more from their physical capability than their understanding of what to do. One United Kingdom participant considered seated leg kick to be the most difficult movement because of the weakness of his quadriceps (UK009, 80 years, low function, female); one Taiwanese participant considered standing knee bend to be the most difficult movement as her hamstring was weaker (TW038, 79 years, low function, female). Regarding Tai-chi snacking movements especially snake creep through the grass was considered to be the most difficult movement because of its squatting posture which could cause muscle soreness. Participants with poorer balance ability found single leg movements to be difficult:

The snake creeps. I always found that difficult when I was doing Tai-chi anyway. I could not squat down because I have stiff hips and back so that’s quite a challenging one to do. It put pressure on my knees. but I could feel my muscles working [TW011, 70 years, high function, female]

Standing on one leg and front heel kick… it is a question of the balance. The snake creep defeated me. The snake got away I think [UK010, 77 years, low function, male]

A clear and simple description of exercise instruction, good communication, and encouragement are important and may increase the exercise participation (Flegal et al., 2007; Sjösten et al., 2007). Accordingly, participants felt that they need more instructions and continuous feedback on Tai-chi snacking programme. Most participants said that it would be nice and more motivating to go to a class or have tutorial to learn Tai-chi:

I think that [a tutorial] is always good for motivation. But I think with the Tai-chi as I explained, because it is something that we’re not familiar with… that kind of movement I think that would be very useful [UK014, 71 yeaear, high function, female]

I think it is quite hard to learn Tai-chi from watching the video and from reading the instructions you’ve sent to us together with the photographs… Having your feedback straightaway, I think, would be a big improvement. I think possibly you need to be with a teacher in the room who watches you and says '‘move your hands to the left’‘, or whatever it might be. The Tai-chi, I’d be quite happy to do it, but I need to go and do a course with an instructor [TW003, 79 years, high function, female]

Finally, participants made recommendations for adding upper body movements in exercise snacking programme and simplifying Tai-chi snacking movements which they thought would be better for novices and avoid the feeling of frustration and improve self-efficacy.

I think probably to maintain a certain amount of strength of muscles, you need to do that sort of thing to use all the bodies, not just legs. We need to do something for the upper body. Because when you get older, your shoulders get very stiff [UK024, 72 years, low function, female]

The exercise snacking would have to be much more strenuous and the Tai-chi much easier [UK013, 79 years, high function, male]

Overall, participants found that the snacking exercises were convenient, particularly the fact that no equipment was necessary, and it can be done anywhere anytime. The short bout snacking format was considered to be attractive and motivational. However, concerning coherence, understanding and execution, exercise snacking was more acceptable. For new audiences, making Tai-chi snacking easier to master initially and breaking it down into several levels, and having some exercise snacking movements for upper body would be more acceptable.

### 3.6 Ethicality: the extent to which an intervention aligns with participants’ values

Participants reflected on the role that exercise played day to day, the meaning and value they attributed to it and how it came to ‘fit’ within the push and pull of their lives as well as in relation to their age and fitness levels. Most participants found the snacking exercises were appropriate and essential for people in their age group. They found it easy to do the snacking exercises at home. Participants commented that the organization of the programme, with its specified duration and type of exercises was helpful and suitable for older people:

I found it very helpful. I felt that it [snacking exercises] was something very relevant to people as they get older… I am losing my fitness. But I want to keep it as good as possible for as long as possible to be able to enjoy myself. And I think this is definitely helping… Introducing to five somewhat basic exercises in each of the two disciplines is a great idea for older people [UK002, 78 years, low function, male]

Nelson et al. (2004) found that a home-based training with minimal supervision was safe and feasible for older adults. Similarly, some of the participants in this study expressed that snacking exercises are somehow safer than traditional exercise training methods and gym exercise machines which for some people are stressful and the risks of getting injury would be higher. However, a small number of participants felt pressure, even pain on their knees while doing exercise snacking movements compared to Tai-chi snacking movements. Studies have shown that unpleasant experience and negative self-perception (i.e., feelings of joint pressure and fear of getting injured) would affect exercise participation and adherence (De Groot and Fagerström, 2011; Reynes et al., 2019). These factors should be considered more carefully when designing exercise programmes in future:

I think for older people, because their knees are not so good. It (exercise snacking) puts pressure on the knees. It is better to do the other exercises which are more gentle on the knees in order to warm up… Tai-chi has greater depth. I like the fluidity and the kind of being on a bended knee. I think it is less pressure on your knees. It is very good for balance and core strength [UK014, 71 yeaear, high function, female]

A few participants thought that some movements (i.e., calf raises, seated leg kick, single whip, snake creep through the grass) would be too difficult for people aged over 80 or for those who have osteoarthritis, joint replacement, and/or musculoskeletal injury history, therefore simplifying particular movements and creating a progressive snacking exercise programme are necessary and could be more useful and helpful for more diverse populations:

As you get older, your feet become a little bit arthritic. And so doing that standing on your toes (calf raises) can be a bit painful [UK014, 71 yeaear, high function, female]

I think the balance is a bit demanding (Tai-chi snacking). I would have thought it would be very difficult for people who are older than me [TW018, 69 years, high function, female]

I think these movements are suitable for older people. However, it would be inappropriate for people with special sport injuries or degenerative joint disease. Simplifying the movements would be better regarding the safety of doing these snacking exercises at home [TW014, 68 years, high function, male]

For a small number of participants, it was hard to fit the exercises in with their household chores, specifically more Taiwanese female participants reported this to be an issue in their busy life. Time for physical activity is an important consideration but time is also a gendered issue (Festini et al., 2019). Some female participants in this research highlighted that their various commitments everyday meant their lives felt hectic and busy. Analysis of their comments suggests that they have taken on much of the household work as well as childcare responsibilities. This unpaid labor is often gendered in terms of its uneven distribution onto women and reflective of cultural context and values too. For instance, this participants’ experiences are constituted by the patriarchal early Chinese cultural values that infuse Taiwanese lives and subjective experiences (Fuligni, 1998) of women living busy lives and therefore having little room for physical activities and exercise:

I found it hard to do the exercises every day. At home. you’ve got lots of distractions. I have so many things to do… I have to take care of my grandchildren, go grocery shopping, attend community events… I could not do your programme every day in this week and I do not think I’ll do it in the future. I’m too busy [TW006, 70years, high function, female]

Furthermore, Tai-chi ethos is closely related to traditional Chinese Taoist culture (Su, 2013). Tai-chi, rooted in the Taoist tradition (the Chinese religious-philosophical movement), is supposed to be aligning more to Asian beliefs, and Taoism values. However, we have not found differences between the United Kingdom and Taiwanese participants when it comes to religious and cultural identities. The findings are consistent with those of Brown (2016), who stated that ‘Tai-chi intermingles religious and holistic forms of spirituality without contradiction.’ (p. 317).

In addition, when asked to feedback the exercise instructions participants suggested that it would be much easier for them to learn and follow the movements if the video instruction could make to be mirror image or film from the back, and take them through a full minute of one movement, particularly Tai-chi snacking movements.

Maybe because of the different sides, not the mirror image does not make it easy. I guess it would be easier for me to do these five movements if the video took you through 1 minute of one exercise [UK028, 69years, low function, female]

The video instruction is helpful, but I would say with the Tai-chi, the only difficulty was initially when you said ‘‘go to the left’‘, you then do the opposite… Your video was face on. I got confused as to whether I should be doing the same leg or mirroring you [TW008, 67years, high function, female]

Given these points, participants found the short bout exercise and Tai-chi snacking programmes to be convenient and appropriate concerning the perceptions of ageing. In terms of gender identity, some Taiwanese female participants considered themselves to be too busy to do the snacking exercises impacted by their personal beliefs and cultural background. Nonetheless, we need to modify some specific movements, provide more options given individuals’ fitness levels and physical conditions for targeting the right groups, and perhaps create progressive exercise programmes in the future.

### 3.7 Self-efficacy: the participants’ confidence of the ability to perform the behavior required to participate in the intervention

Self-efficacy is a moderating factor regarding exercise participation and adherence (Luszczynska et al., 2011). Studies have shown that the sense of achievement and enjoyment may enhance the engagement in physical activities (Lee et al., 2007; Lewis et al., 2016). Similar findings were found in this study. In most participants, feelings of satisfaction, enjoyment, and being energized were common experiences that can help explain their willingness to do the snacking exercises. A small number of participants also found that they were better equipped and more confident exercising by themselves. However, some participants reported that they had lower confidence in performing Tai-chi snacking, specifically those participants who were considered low on physical function. For these participants there was more hesitancy about engaging with the snacking protocol and for some they were more fearful:

I had a lot of energy. I felt satisfied after doing the programmes. If I’ve done well, I think, ‘oh, well, that’s good.’. I felt better if I achieved it. It is good to make the brain do different things [UK030, 78years, high function, female]

Tai-chi, I can’t practice it very well. And my balance does not help. If you’ve got your balance, I can understand it is quite relaxing [UK010, 77years, low function, male]

A small minority of participants found Tai-chi snacking frustrating since they could not receive immediate feedback and were not sure if they did the movements properly.

The trouble is, I forgot some of the moves because there’s five moves. And to remember all the angles. I just could not get that right. I think if we’d been in a class where you’ve been here, and you’d said, ‘Do this snake creep through the grass’, and got us doing it four or five times until we really got it. That would have been fine [TW024, 70years, high function, female]

Few participants expressed that they would not carry-on doing Tai-chi snacking, whereas most participants were willing to keep doing exercise snacking over time in the future. This notion would be supported by previous studies indicating that self-efficacy is influenced by personal affective and behavioral factors, and has a direct correlation with exercise participation and adherence (Oman and King, 1998; McAuley et al., 2003; McAuley et al., 2011).

I certainly want to continue doing the exercise snacking. However, I do not think I’ll carry on doing the Tai-chi snacking. With the fact that I think a lot of older people would have trouble with Tai-chi [TW031, 78 years, high function, female]

I think the exercise snacking I will continue to do after your study, but Tai-chi, I think unless I’m really sure that I know what I’m doing. I think it’d be more difficult. I’ll do the exercise snacking. Tai-chi, at the moment, that would be impossible. I think I need more feedback [UK020, 77 years, low function, male]

Accordingly, participants felt their physical function to be better than they thought and had been more confident exercising on their own, whereas some participants particularly with low physical function thought themselves to be not fit enough, and experienced difficulties and felt unsafe when practicing Tai-chi snacking. Thus, to avoid lack of confidence and improving the engagement in participating exercises, it is important to consider individuals’ physical function when designing exercise regime, and finding ways of supporting the initial engagement and mastering the movements via in person or, perhaps, video instruction and support.

Above all, there were not many differences between the United Kingdom and Taiwanese participants when it comes to the capability of executing the exercise and Tai-chi snacking programmes and the confidence in the ability to perform them. Both United Kingdom and Taiwanese participants considered the snacking exercise easy and convenient to do at home and they did not have to sacrifice doing something for our exercise programmes. Moreover, the perceived effort did not vary from United Kingdom to Taiwanese participants even if Tai-chi is a relatively widespread exercise form in Taiwan. Altogether, we have not found many differences between the Taiwanese and United Kingdom participants in the burden dimension, the opportunity costs, the intervention coherence, as well as the self-efficacy dimension. Furthermore, we have not found differences between the United Kingdom and Taiwanese participants when it comes to religious and cultural identities although Tai-chi is supposed to be more relative to Asian beliefs, and Taoism values as it is based on the Chinese religious philosophy (Wang et al., 2000; Brown, 2016).

However, some Taiwanese female participants reported that living busy lives, and having much household work and childcare responsibilities prevented them from being able to do physical activities and exercises. This gender identity affected by different cultural backgrounds, especially the influence of the patriarchal early Chinese cultural values in Taiwan, is an interesting cultural finding in our study and may warrant further exploration and attention in the context of physical activity and exercise promotion amongst this demographic. We also found that more United Kingdom participants preferred Tai-chi snacking because of its elegant and relaxing movements regarding participants’ feelings and attitudes towards the snacking exercises. United Kingdom participants also expressed their interest in learning the origins and values of Tai-chi as this represents a new format of exercise for them. Some United Kingdom participants mentioned that understanding the spiritual-cultural origins of Tai-chi (such as the concepts of Yin and Yang, and qi) may enhance their experiences of Tai-chi snacking, whereas for Taiwanese participants this was more familiar. Given these points, personal affective attitude and different cultural backgrounds may affect exercise participation; yet how participants feel about the snacking exercise did not appear to differ between the backgrounds and nationalities included in the present study.

## 4 Conclusion

The Theoretical Framework of Acceptability analysis has provided us with a useful lens for examining the thoughts and feelings of Taiwanese and British older adults towards exercise and Tai-chi snacking providing novel understanding of how this format of exercise could be implemented into older adults’ homes. Taking a deep dive into the different dimensions of acceptability has shone a light on the strengths of this novel format of muscle strength and balance exercise that make it an attractive proposition for older adults, such as the convenience and appropriateness of performing 10-min of exercise per day, and belief in its utility in improving physical function. We have also identified ways that it might be enhanced for certain people to improve uptake and engagement, such as the need to provide more guidance to improve confidence and mastery of Tai-chi movements for people with lower physical function, and opportunities for more challenging, whole body, exercises to avoid tedium for those with higher function.

In this study we found very little difference in the perspectives of British and Taiwanese participants towards the exercise and Tai-chi snacking protocols. The snacking protocols are designed to deviate from traditional class-based or leisure setting provision by offering participants a convenient mode to engage in strength and balance training in a safe and comfortable way. In our study, Tai-chi snacking was seemingly more favorable in specific dimensions such as effectiveness and lack of burden, whereas when it came to coherence and understanding and being able to execute, exercise snacking was more acceptable. Tai-chi, which harnesses different skills, focuses on breathing, and is more about flow, connection, and meditation, and less mechanical and functional, connects participants to different cultural values. Although Tai-chi is rooted in the Chinese religious tradition, cultural or religious alignment of our study participants seemingly mattered little in their judgement of acceptability. In fact, UK-based participants seemed to express stronger preferences for the elegant and relaxing movements of Tai-chi snacking and a desire to learn more about the origins and values of this enjoyable new form of exercise. It should be noted that in a snacking format aspect of traditional Tai-chi delivery may be lost such as the prolonged, instructor-led, meditative components, and comparisons to long-format Tai-chi cannot be made. However, as the goal of our reduced dose exercise is to support initial engagement of muscle strength and balance exercise amongst older adults doing nothing rather than a long-term solution, it was encouraging to witness acceptability. Accordingly, these findings offer encouragement to practitioners who may be seeking new ways to engage older adults in exercise and demonstrate the potential of deviating from instrumental exercise promotion that focuses purely on physical health in a simple and inclusive format.

The strengths of the current work include the in-depth interviewing of two cultural backgrounds and of varying functional status, which enabled us to compare and contrast the experiences of our new exercise protocols in a heterogenous population. This is also the first study, to our knowledge, that has explored the acceptability of a snacking model of Tai-chi. There are of course inherent differences in the strength exercise and tai-chi snacking modalities that were examined in this study, such as the number of repetitions, the pace at which participants were asked to perform the respective movements. Accordingly, direct comparisons between the two are not necessarily permitted in terms of their intensity, which may have impacted perceptions of acceptability. A further limitation of our study design is the short exposure to the snacking protocols (3 days each) meaning we have only evaluated participants initial judgement, and so the long-term acceptability and understanding if perceived interest would translate to sustained engagement and effectiveness remains unknown. Also, without using quantitative methods and a larger sample size we cannot determine the external validity our findings on the acceptability of exercise and Tai-chi snacking as representative to a wider sample of target older adults. Based on the findings, and to address these limitations, a longer-term experimental study of the efficacy of exercise and Tai-chi snacking for improving physical function would be required to understand how well a heterogenous older adult population adheres to these exercise and Tai-chi snacking protocols in the modified, shortened format, and their impact on health and wellbeing outcomes.

## Data Availability

The raw data supporting the conclusion of this article will be made available by the authors, without undue reservation.
