# Supplementary material for: The acceptability of homebased exercise snacking and Tai-chi snacking amongst high and low function UK and Taiwanese older adults
Source: Front Aging. 2023 Aug 1;4:1180939. doi: 10.3389/fragi.2023.1180939 (PMC10428172; doi:10.3389/fragi.2023.1180939)
Supplement: Supplementary file 1 [file DataSheet1.PDF]

Supplementary file A: exercise instructions

Exercise snacking instructions

What is exercise snacking?

This is a method of structuring exercise into short bouts. We are asking you to try this short exercise snacking routine on 3 days this week, which will include 5 minutes of exercise and 1 minute of rest between each exercise. The exercises themselves are safe for the home and don't require a warm-up before starting. The exercises do not require any sports clothing or equipment; just a kitchen chair.

How is the exercise performed?

During each exercise bout, spend one minute performing each of the five exercises detailed below. Complete repetitions of each exercise at a self-selected pace that is comfortable to maintain for the full minute, with the aim being to complete as many repetitions as possible in that minute. If your legs begin to feel unduly sore during the any of the exercises, you may of course stop that exercise at that time. Take one minute between each exercise to rest.

What is the exercise snacking routine?

- **Sit-to-stand:** This exercise is simply repetition of rising from an up-right seated position on a kitchen chair, and then returning to the seated position. Try to keep your arms folded across your chest to avoid using your arms to aid rising from the chair, making sure that your legs are doing the work. We suggest that this always be the first exercise performed in the routine, and recording the number of sit-to-stands performed in the minute each time lets you track your progress.

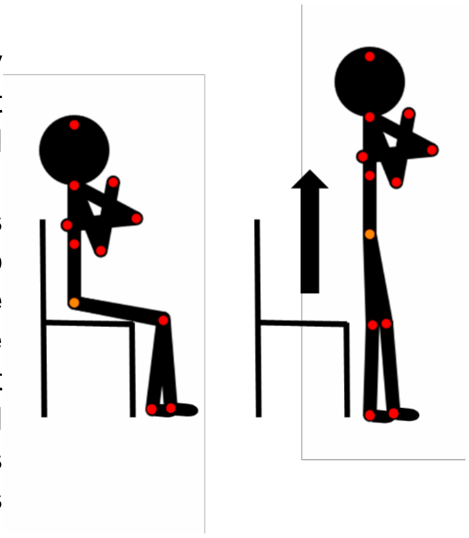

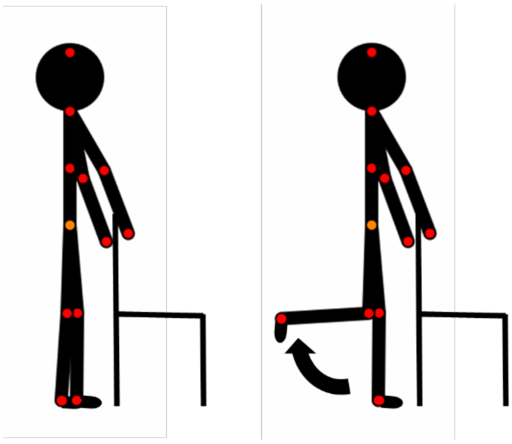

- **Standing knee bends:** Standing up-right and holding onto something stable for balance if needed, raise one foot at a time by bending your knee to roughly a right angle. Then return that foot to the floor, regain your balance if necessary, and repeat with the opposite leg.

- **March on the spot:** Standing up-right, put your arms out in front of you and your hands roughly at waist height, raise one leg up by bending your knee and hip as per the diagram. Aim to get your thigh as close to horizontal as possible, with the top of your thigh touching your hands, then return to a standing position, regain balance if necessary, and repeat with the other leg. If you struggle with balance, you can hold onto something stable like the back of a chair with one hand.

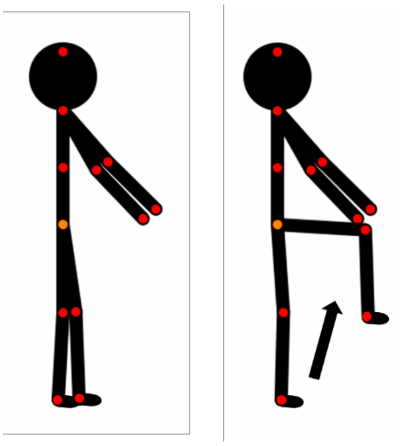

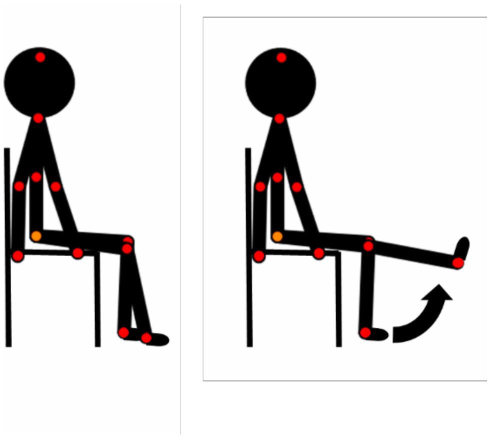

- **Seated leg kicks:** Sitting up-right in a chair, straighten your knee to raise your leg out in front of you at a controlled speed, and then return that leg to the starting position, and repeat using the other leg. If you have quite long legs, it may help to place a rolled up towel under your thigh to raise your knee a little higher in the start position.

- **Standing calf raises:** Start with your feet flat on the floor and rise up onto your tip-toes as high as you can, then return to the start position with feet flat on the floor. It is advisable to hold onto something stable like a chair, table, or door frame to maintain balance. Perform the raises on both legs at the same time, and try to complete as many as you can in a minute, and then rest for a minute.

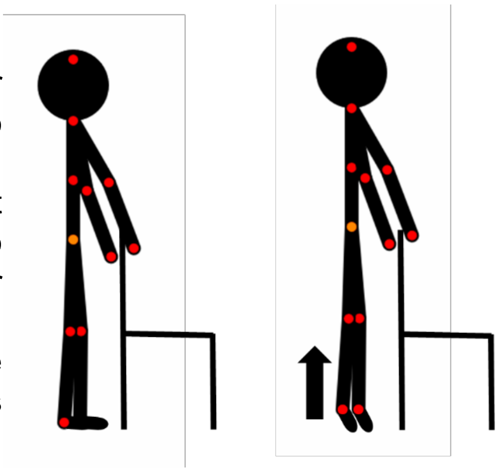

What are the risks of performing the exercise?
As with any exercise, you are likely to feel tired towards the end of the session and in the time straight afterwards. However, the principle of exercise snacking is that the bouts should not be excessively demanding, with each exercise of the regime performed for only one minute at a self-selected repetition speed. The primary risk when performing the exercise is loss of balance during the standing exercises. This risk can be mitigated by performing the movements at a controlled, and by holding onto a stable object such as a chair, table, or door frame. This exercise regime has been designed specifically to avoid any cardiovascular load, as might be experienced during running. If your legs begin to feel unduly sore during the any of the exercises, you may of course stop that exercise at that time.

Tai-chi instructions

What is Tai-chi?

Tai-chi combines deep breathing and relaxation with flowing movements. The five main lower limb movements are selected for this routine. We are asking you to try this short Tai-chi routine on 3 days this week, which will include 5 minutes of exercise and 1 minute of rest between each exercise. The exercises themselves are safe for the home and don't require a warm-up before starting. The exercises do not require any sports clothing or equipment; just a kitchen chair if you feel you need extra support.

How is the exercise performed?

During each exercise bout, spend one minute performing each of the five exercises detailed below. Complete repetitions of each exercise at a self-selected pace that is comfortable for you to maintain for the full minute, with the aim being to complete them as accurately and smoothly as possible (based on correct posture and proper alignment). If your legs begin to feel sore during the any of the exercises, you may of course stop that exercise. Make sure you take one minute of rest between each exercise.

What is the Tai-chi 5 main movements routine?

- **Cloud hands, going left :** Standing with your knees slightly bent, feet shoulder width apart and facing forward, shifting your body weight from the right leg to left leg with the hands crossing from right to left (with left palm facing you at shoulder height and right palm facing the floor at waist height). After shifting your body weight to left leg completely, take a small step to bring your right foot back in and change arms so the right is on top, and left at waste height. Shift your body weight back to the right leg, and repeat the movement. We suggest that this always be the first exercise performed in the routine.

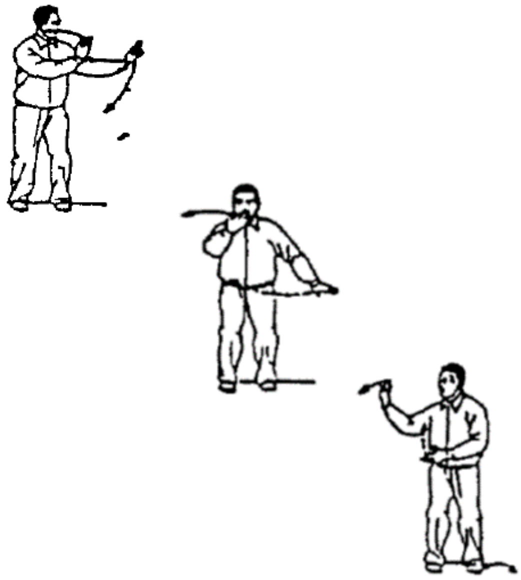

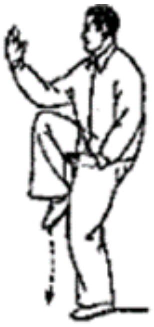

- **Stand on one leg:** Start in a standing position with your hands by your side. Shift your weight to left leg; lift the right heel off the ground and then raise your right leg off the floor and right arm with hand facing inwards in a slow steady movement. Keep your

left knee slightly bent and left arm by your side. Aim to get your right thigh as close to horizontal as possible, then return that foot to the floor, regain your balance if necessary, and repeat with the right leg for 30 seconds after doing stand on left leg for 30 seconds. If you struggle with balance, you can hold onto something stable like the back of a chair.

- **Single whip:** Hook your fingers and thumb of the right hand in a slight closure and place the left hand nearer to your face with palm facing yourself; shift back and lift the left heel. Try to keep your weight on single leg stably then shift your body weight from your right leg onto your left leg slowly and then take a step out with your left leg (pushing with the right leg when you get beyond halfway). Turn your hips and open your arms outward at the same time, making sure that your legs are doing the work in a slow, controlled motion.

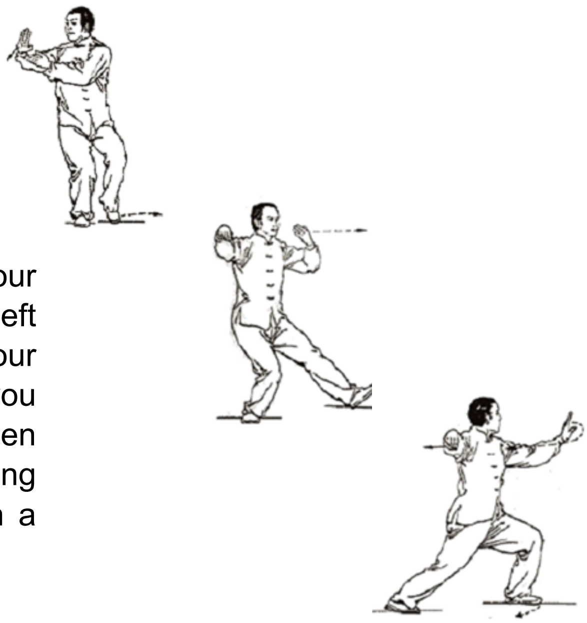

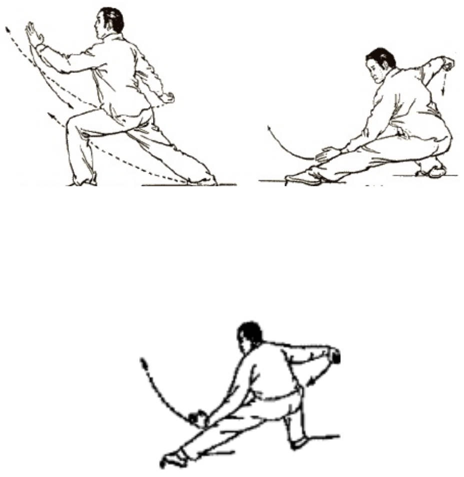

- **Snake creeps through the grass:** Starting from the final posture of the Single whip exercise, turn out the right toe (so your feet are at 90 degrees) and open your hip joint. Shift back and sit into the right leg, slowly drop down as low as possible with left hand crossing near your ankle. Then push off the right leg with left leg bending progressively, and shift your body weight to the left leg, go forward with left hand end at shoulder height. The most important thing is keeping your spine as straight as possible, not how low you go.

- **Front heel kick:** From a standing position with your hands cross, sit gradually into the left hip and lift the right heel. Try to keep your weight on left leg and remain stable while raising your right leg with your knee bent, then kicking the right leg forward slowly and open the arms outward at the same time. Aim to kick your leg (slowly) in a controlled manner as high as possible, then slowly return that foot to the floor. Regain your balance if necessary and repeat with the left leg kicking for 30 seconds after doing right leg kicking for 30 seconds. If you struggle with balance, you can hold onto something stable like the back of a chair with one hand.

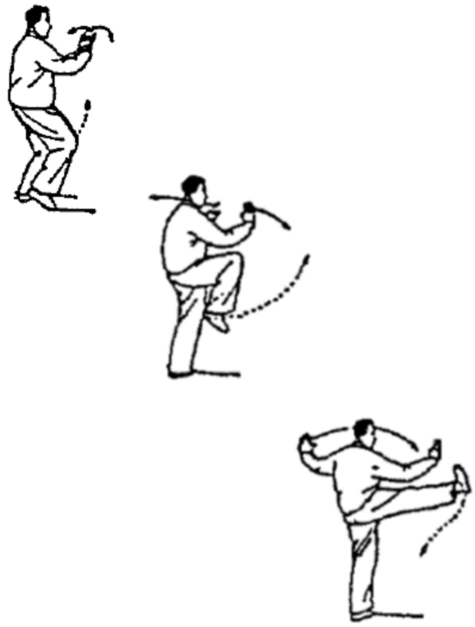

What are the risks of performing the exercise?

As with any exercise, you are likely to feel tired towards the end of the session and in the time straight afterwards. However, the principle of Tai-chi is that the movements should not be excessively demanding, with each exercise of the routine performed for only one minute at a self-selected repetition speed. The primary risk when performing the exercise is loss of balance. This risk can be mitigated by performing the movements at a controlled speed, and by holding onto a stable object such as a chair, table, or door frame. This exercise regime has been designed specifically to avoid any cardiovascular load, as might be experienced during running. If your legs begin to feel overly sore during the any of the exercises, you may of course stop that exercise at that time.
